# FP2020 and FP2030 Country Commitments: A Mixed Method Study of Adolescent and Youth Sexual and Reproductive Health Components

**DOI:** 10.9745/GHSP-D-24-00223

**Published:** 2024-10-29

**Authors:** Asantesana Kamuyango, Shreya K. Arora, Laura Raney, Ahmed K. Ali, Venkatraman Chandra-Mouli

**Affiliations:** a Department of Sexual and Reproductive Health and Research, UNDP/UNFPA/UNICEF/WHO/World Bank Special Programme of Research, Development and Research Training in Human Reproduction, World Health Organization, Geneva, Switzerland.; b International Programs, Population Reference Bureau, Washington, DC, USA.; c FP2030, United Nations Foundation, Washington, DC, USA.; d Formerly with the Department of Sexual and Reproductive Health and Research, UNDP/UNFPA/UNICEF/WHO/World Bank Special Programme of Research, Development and Research Training in Human Reproduction, World Health Organization, Geneva, Switzerland.

## Abstract

**Introduction:**

Family Planning 2020 (FP2020) was established in 2012 with the goal of expanding contraceptive access. By 2020, 46 countries had made commitments to FP2020. A sustained focus on adolescents and youth (AY) began in 2016. During the commitment formulation process, substantial support was offered to countries to develop AY commitments based on sound data, research evidence, and programmatic experience. This study assesses how country commitments under FP2020 and FP2030 have evolved over time with respect to improving attention to and focus on the needs of adolescents and youth sexual and reproductive health (AYSRH).

**Methods:**

We analyzed the content of FP2020 and FP2030 country commitments focusing on AY (aged 10–24 years) using a scoring guideline we developed to measure the AY commitments in terms of completeness, clarity, and quality.

**Results:**

This analysis shows that FP2030 commitments better articulate strategies and activities to reach AY with contraceptive information and services when compared to FP2020 commitments.

**Conclusion:**

FP2030 commitments are stronger in some areas on AYSRH, such as commitment to establish national or local policies, strategies, and guidance for AY programming, specifying the target audience of the AY commitment, and partnering with AY or youth-led organizations in commitments. However, more work remains to be done by countries to dedicate a budget for achieving AY objectives, including measurable targets for monitoring progress, identifying and addressing the root causes that impact AY access to and use of contraception, including child marriage and gender-based violence, and reducing financial barriers to access contraception.

## Abstract (precis)

While FP2030 commitments better articulate strategies and activities to reach adolescents and youth with family planning (FP) information and services compared to FP2020 commitments, gaps remain. To achieve the Sustainable Development Goals, countries should continue to invest in creating and funding comprehensive FP commitments that meet the sexual and reproductive health needs of adolescents and youth.

## Teaser

**Key Findings:**

- Adolescent and youth sexual and reproductive health (AYSRH) content is stronger in the FP2030 country commitments compared to the FP2020 country commitments.
- However, the AY commitments still lag in terms of having a specified budget for adolescent and youth (AY) family planning (FP), specifying measurable targets for monitoring progress of their AY objectives, identifying and addressing root causes that impact AYFP, and clearly articulating their commitment to reducing financial barriers.

**Key Implications:**

- The AY population continues to grow, as does the need for knowledge of and access to contraception.
- Policymakers and program managers should continue to strengthen their policies and programs and increase their budget allocations for AYSRH to meet the needs and fulfill the rights of adolescents and youth.

## INTRODUCTION

Globally, adolescent birth rates have declined from 52 births per 1,000 girls in 2010 to 42.5 in 2021.^1^ South Asia has made the most progress in reducing early childbearing, with the adolescent birth rate dropping from 49.6 births per 1000 girls in 2010 to 29 births in 2021.^1^ Sub-Saharan Africa has also made notable progress, with the adolescent birth rate declining from 117.7 births per 1,000 women in 2010 to 101 births per 1,000 girls in 2021. However, because sub-Saharan Africa has historically had a high adolescent birth rate, its levels continue to be high compared to other regions, with large disparities within the region.^1^ Although there has been notable progress in addressing adolescent pregnancy and childbearing, the rate of improvement has been relatively slow, with a decline of approximately 3 percentage points per decade over the past 6 decades.^2^^,^^3^ There are sound public health, economic, and human rights reasons to address these inequities and improve access to and uptake of contraception by adolescents.^4^

Between 2010 and 2020, the proportion of adolescent girls globally aged 15–19 years whose needs for family planning (FP) were satisfied by modern methods rose from approximately 49% to 60%.^5^ Although there has been progress globally, both the levels and trends have been uneven across regions. Latin America and the Caribbean and Eastern Europe and Central Asia regions had the highest levels of demand for FP satisfied by modern methods (more than 70%) in 2020, compared to 88% in North America.^5^ Although South Asia, sub-Saharan Africa, and the Middle East and North Africa have observed consistent increases in the proportion of adolescent girls having their demand for FP satisfied by modern methods, the percentages remain lower than 50% in these regions. Overall, there has been an increase in the aggregate level of modern contraceptive use in low- and middle-income countries among adolescents aged 15–19 years from 17.8% in 2000–2006 to 27.2% in 2013–2017 and adult women aged 20–34 years from 30.9% in 2000–2006 to 40.3% in 2013–2017.^6^

## EVOLVING COMMITMENT TO FAMILY PLANNING

As Bongaarts and Sinding, 2 authorities in FP, recalled, between the 1960s and the mid-1990s, many countries established voluntary FP programs to respond to the challenge of rapid population growth.^7^ These efforts were championed and supported by international organizations and funding agencies. Some of these programs were designed and executed better than others, but overall, they achieved tremendous success. As noted by Lapham and Mauldin, they “contributed substantially to increased contraceptive use and to declines in fertility.”^8^

Commitment to FP, both financially and politically, peaked around the 1994 International Conference on Population and Development (ICPD) in Cairo.^2^ However, from the mid-1990s, both the championship and the funding for FP programs declined on the basis of a number of misconceptions and false arguments.^9^ Some misconceptions about FP programs included beliefs that they had minimal impact on fertility, that declining fertility rates meant they were no longer needed, that the AIDS epidemic made FP programs unnecessary, that they were not cost-effective, and that they either exploited women for population control or were coercive.^10^

To respond to this challenge, the United Kingdom government, the Bill & Melinda Gates Foundation, United Nations Population Fund, and U.S. Agency for International Development convened a global FP Summit in London in 2012. At this Summit, Family Planning 2020 (FP2020), a groundbreaking partnership formed to make affordable, lifesaving contraceptives, information, services, and supplies available to an additional 120 million women and girls in the world’s poorest countries by 2020, was created. The FP2020 partnership’s vision was to ensure women in these countries could have the same freedom to access FP services—without coercion, discrimination, and violence—as women in high-income countries. Governments, civil society, and communities were called on to tackle the many barriers that prevent women and girls from using FP, such as a lack of contraceptives, lack of money, and lack of support from their husbands.^11^

The FP2020 country-engagement process began with informal exchanges between FP2020 and the ministries of health, leading to a country making a formal FP commitment. The FP2020 Secretariat then linked the committing country to a wide network of partners and donors. Then, focal points representing the government, civil society, and key partners worked together with the FP2020 Secretariat to drive progress toward the country’s FP2020 objectives and create their country “costed implementation plans,” which covered the major FP program elements. The focal points attended regional focal point workshops with partners and the FP2020 Secretariat every 18 months to develop actions for acceleration plans—short-term plans that closely aligned with the costed implementation plan.^12^

Between 2017 and 2020, the partnership supported increased attention to adolescents and youth (AY) through facilitating youth participation and programming and providing enhanced technical support to countries to strengthen programs and services.^13^ Events leading up to this focus on AY included the 2016 FP2020 reference group meeting and the 2017 Second London Summit on FP.^14^^,^^15^ In late 2018, FP2020 jointly launched the Global Consensus Statement on Meaningful Adolescent and Youth Engagement with the International Youth Alliance for Family Planning and the Partnership for Maternal, Newborn & Child Health. An emphasis on meaningful AY engagement was included in the regional focal point meetings in Anglophone and Francophone Africa (including Haiti) and Asia. At these meetings, the focus was on supporting countries in developing sound policy, program, and budget commitments and discussing the translation of these country commitments into action to meet the needs and fulfill the rights of AY. The Human Reproduction Programme, housed in the World Health Organization’s (WHO) Department of Sexual and Reproductive Health and Research, was actively involved in these regional meetings by having staff members conduct sessions and work with the FP2020 AY team to respond to country requests for technical assistance. In addition, by the end of 2019, youth focal points were appointed in each commitment-making country where they joined the government, civil society, and donor focal points.

In 2021, when FP2030 was created as a transition from the FP2020 partnership, HRP staff worked with the FP2030 team to jointly develop and disseminate guidance on how to develop strong and actionable commitments to AY sexual and reproductive health (SRH).^13^ As the commitments were developed, HRP and FP2030 staff worked together to review and provide feedback on the content of the draft country commitments. In some cases, follow-up meetings were held with country teams to provide them with recommendations based on sound data, research evidence, and programmatic experience.

FP2030 learned from FP2020’s country commitment process and now focuses on stronger country ownership. FP2030’s revised process also offers guidance on incorporating a rights-based approach, highlighting key areas, including AYSRH, postpartum and postabortion FP, financing, emergency preparedness, method choice, and strengthening supply chains.^16^ FP2030’s new model also emphasized the importance of accountability mechanisms by fostering strong collaboration with civil society and youth partners.^13^ Finally, in both FP2020 and FP2030, country governments shared the final commitment with key stakeholders, including youth and civil society representatives, to achieve formal validation.^17^

This article examines the country commitments of FP2020 (2012–2019) and FP2030 (2021–2024) to ascertain if the commitments’ content reflects the increased emphasis on and sustained effort to support countries to develop AY commitments based on sound data, research evidence, and programmatic experience. Although some workers believed that time, effort, and resources spent in engaging and advocating with ministries of health and other government officials would lead to stronger policy, program, and budget commitment, which, in turn, would lead to great investment and concerted action, others were less convinced.^18^ Therefore, this analysis was conducted to assess how country commitments to FP2020 and FP2030—developed by ministries of health, with inputs from partners within and outside the countries—have evolved over time with respect to improving access to contraception information and services for AY by asking the following research questions.

1. In terms of AY content, what did each country’s commitment include when examined by the 3 domains of completeness, clarity, and quality (Box)?^19^
2. Did the AY content of each country’s commitment change relative to these 3 domains between FP2020 and FP2030?
3. Overall, in which domains was progress seen over time, and in which domains is further improvement or strengthening needed moving forward?

This analysis was conducted to assess how country commitments to FP2020 and FP2030 have evolved over time with respect to improving access to contraception information and services for AY.

## METHODS AND ANALYSIS

### Data Sources

Between 2012 and 2019, 46 of the 69 priority countries made commitments to FP2020. Of those, 45 countries included an AY component; thus, our analysis includes these 45 country commitments. Burkina Faso was not added for FP2020 analysis because, despite having a commitment, there was no AY content to be analyzed. FP2030 commitments began in 2021 and are ongoing. We included the 33 countries whose commitments were available on the FP2030 website as of February 29, 2024. All 33 country commitments include AY; thus, they are included in our analysis. Both the FP2020 and FP2030 country commitments were retrieved from the FP2030 website.^20^^,^^21^ There were 31 countries that made commitments to both FP2020 and FP2030 and included an AY component. We conducted analysis for countries with commitments in either 2020 or 2030, as well as the 31 countries with commitments in both periods to ensure the validity of the results.

For all 45 FP2020 and 33 FP2030 country commitments, the portion relating to AY was extracted. The content was then grouped by country and commitment period (FP2020 or FP2030) into 1 of 3 categories: policy, financial, or programmatic. The programmatic category was further subdivided into service delivery and social and behavioral change programs.

### Scoring Guideline Development

In 2021, FP2030 and WHO staff working under the WHO Adolescent and Youth Sexual Reproductive Health and Rights Technical Assistance Mechanism developed a checklist that WHO HRP staff used to review draft country commitments and provide recommendations to FP2030 country focal point teams while developing their AY commitments.^22^ The checklist was informed by the FP2030 Adolescent-Friendly Contraceptive Services Scorecard, which allows an examination of existing country AY policies and the identification of priority areas for countries to adopt and implement evidence-informed AY contraception policies and programs. The checklist included 5 items: (1) integrating AY throughout the FP2030 policy, programmatic, and financial commitments; (2) addressing root causes of high adolescent pregnancy and childbearing, especially among the most vulnerable AY; (3) utilizing evidence-based and High Impact Practices in FP (HIPs) and avoiding investments in approaches that have shown to be ineffective; (4) enhancing capacity in the use of age-disaggregated data; and (5) engaging AY in the commitment-making process. These items acted as the basis for developing the scoring guideline in the current study to score the FP2020 and FP2030 AY components of the countries’ commitments, especially for subdomains for the quality domain.

The scoring guideline was developed in an iterative fashion, with 3 successive versions created that led to the final design. With each version, 2 researchers separately piloted the scoring guideline by scoring the FP2020 and FP2030 commitments of 3 countries. The entire research team then discussed the results and reached a consensus on the domains, items included in each domain, definitions, scoring indicators, and values for each item.

### Domains, Items, and Definitions

The scoring guideline was developed to assess and score each country’s commitment on 3 domains of their AY commitment: completeness, clarity, and quality (Box).^23^^–^^25^ Each domain was defined, the items that comprise each domain were described, some with examples, and each was assigned a score.

BOX Three Domains of Adolescent and Youth Components of Country Commitments to FP2020 and FP 2030

1. **Completeness**–Measures the extent to which a country’s adolescent and youth (AY) commitment includes components on:
  - Policy: establishment of national or local policies, strategies, and developing guidance
  - Programmatic:
    - Service delivery: provide and improve AY services and remove barriers to promote AY access to contraception
    - Social and behavior change: address individual, interpersonal, and community-level factors to enable better AY access to and use of contraception
  - Financial: targeted specifically for AY programs
2. **Clarity**–Measures the extent to which the country’s commitment included information on:
  - Target audience of their objectives (married or unmarried adolescents, boys, men, parents, partners, teachers, or health care providers)
  - Measurable target to monitor the progress of their objectives
3. **Quality**–Measures the extent to which the commitment includes these 6 evidence-based and High Impact Practices in FP (HIPs) determined as best practice in AY programs:
  - Involve AY: defined as partnerships with AY or youth-led organizations; consideration of joint development, launch, and/or implementation
  - Use data-based approaches to determine their objectives (i.e., data disaggregation by age, gender) and evidence-based approaches and HIPs in their objectives: (e.g., integrating adolescent family planning and contraception services, reducing financial barriers to access, and avoiding ineffective interventions or strategies, such as standalone peer education, youth centers, or clinics)
  - Focus on strengthening data quality: enhancing the country’s capacity to collect and use disaggregated data by age, gender; measure the commitments’ progress; or evaluate their objectives
  - Involve multiple sectors within the government to achieve commitment objectives
  - Involve entities other than the governmental sectors (e.g., nongovernmental organizations, youth-led organizations, private sector, donors, or academic institutes)
  - Address root causes that impact AY access to and use of contraception, including gender-based violence, violence against girls and women, child marriage, or girls’ education, and propose measures to address these root causes

### Scoring and Analysis

Within each domain, points were subjectively assigned for each item based on the importance of that item within each of the domains. The item scores within each domain were summed to provide the total domain score. The scores for the 3 domains were then combined to provide an overall score for the country’s AY commitment. The scores assigned to each domain—if each item and subitem was satisfied—were calculated as follows: completeness=3, clarity=1, and quality=9. Thus, when added, the highest possible total score for a country’s AY commitment was 13. If an item was not met, it was scored as a zero. If it was satisfied, it was scored with the score value assigned for the item. More details on the scoring indicators and scores assigned to each item and subitem are provided in Supplement Table S1.

Two researchers independently scored each FP2020 and FP2030 AY country commitment, reviewed the scoring, and resolved any differences. For the purposes of analysis and comparison, domain scores were coded into 1 of the following 2 categories: (1) AY commitments with a domain score that is less than or equal to 50% of the total possible domain score and (2) AY commitments with a domain score greater than 50% of the total domain score. For example, if Mali’s FP2020 AY commitment scored 4 of 9 in the quality domain, Mali was included in the first category. This score coding was also used for the overall score of the AY commitments (Table 1).

**TABLE 1.** Country AY Commitment Domain Score Coding Categories Used in Analysis

**Domain**	**Highest Possible Score**	**Scoring Categories**	
		**1:** ≤**50% of Total Possible Domain Score**	**2: >50% of Total Possible Domain Score**
Completeness	3	≤1.5	>1.5–3
Clarity	1	≤0.5	>0.5–1
Quality	9	≤4.5	>4.5–9
Total	13	≤6.5	>6.5–13

## RESULTS

First, we present analysis results of the 33 commitments on AY FP from FP2030 countries and 45 from the FP2020 countries. Then, we present the results of the 31 countries that had commitments in both FP2020 and FP2030. The details of the countries analyzed for both FP2020 and FP2030 are in the Supplement Table S2.

**FIGURE 1:**
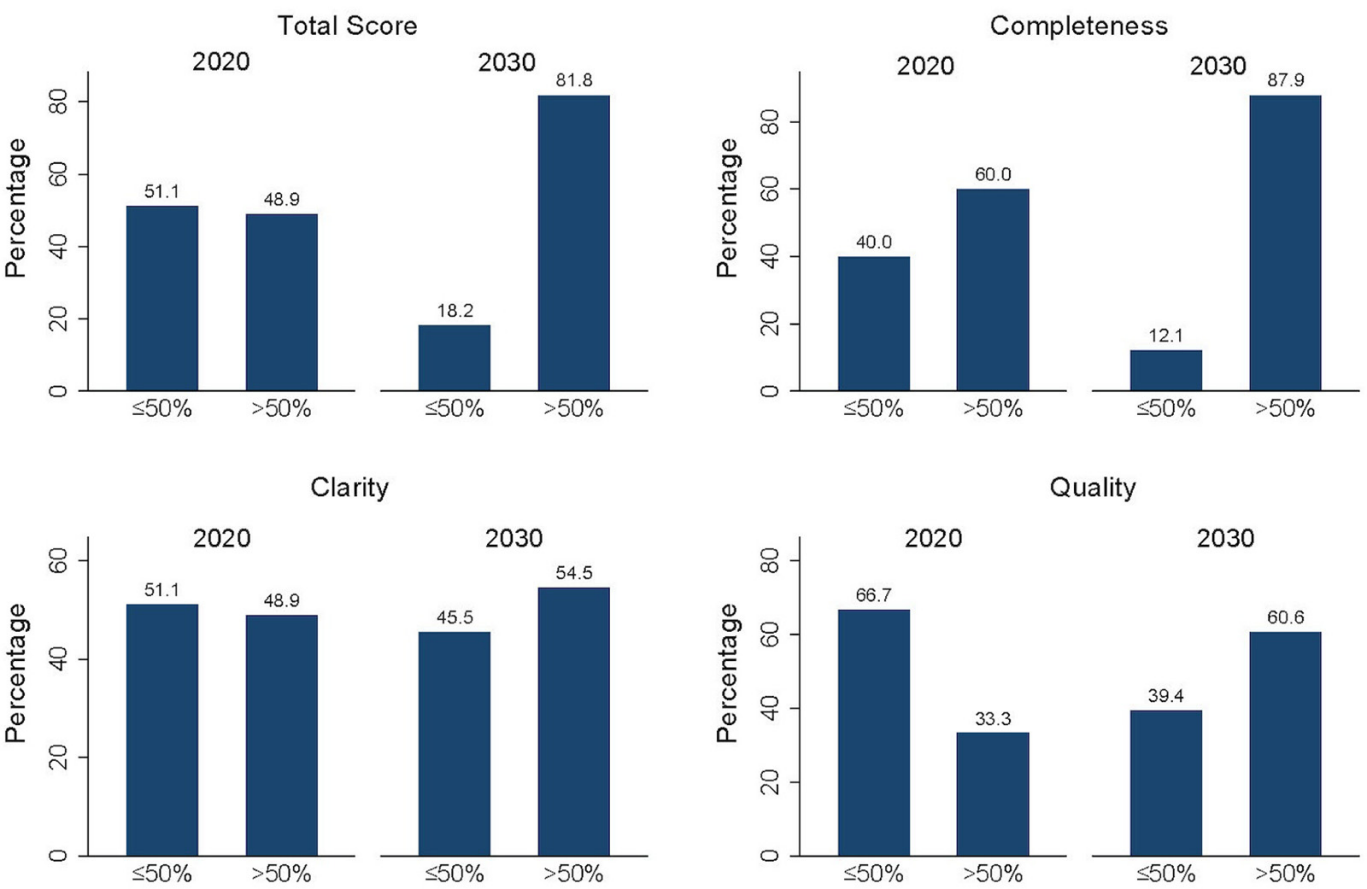
Totals, Completeness, Clarity, and Quality Domain Scores for Country Adolescent and Youth Commitments, FP2020 and FP2030

The combination of scores for all 3 domains provided an overall score for the country’s AY commitment. Overall, only 22 of 45 (49%) of FP2020 AY commitments scored higher than 50%, whereas 27 of 33 (82%) FP2030 AY commitments scored greater than 50%, which shows an increase of 67% (Figure 1 and Supplement Figures S1–S6 for data visualizations). An increase was observed across all domains between FP2030 and FP2020 AY commitments. The greatest increase was observed in FP2030 AY commitments’ quality and completeness, both increasing by 47% from FP2020 AY commitments’ scores, in comparison to their clarity, which only increased by 12%.

An increase was observed across all domains between FP2030 and FP2020 AY commitments.

### Completeness

**FIGURE 2:**
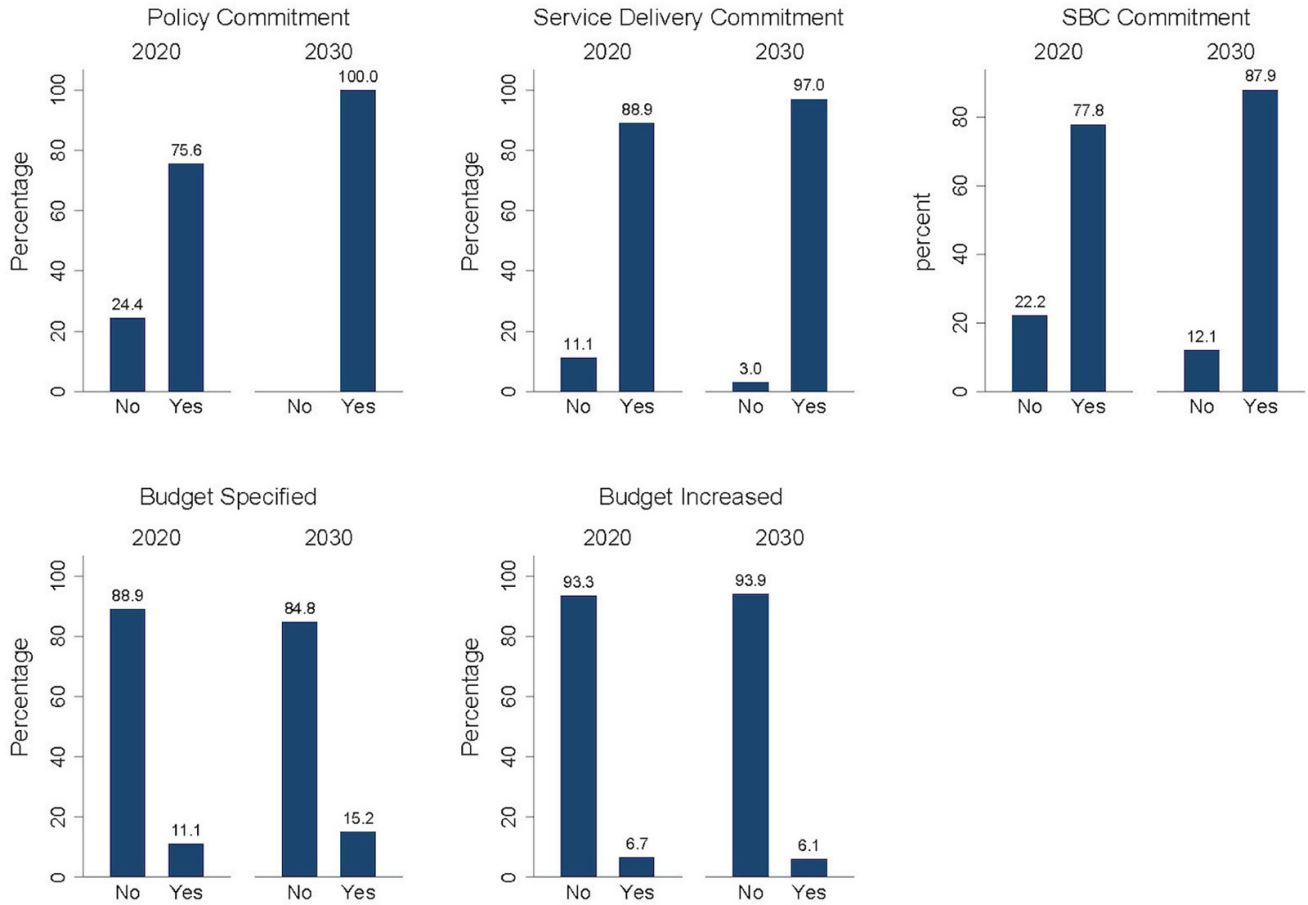
Completeness Domain Component Scores for Country Adolescent and Youth Commitments, FP2020 and FP2030 Abbreviation: SBC, social and behavior change.

The domain scores for completeness show the extent to which AY commitments included policy, programmatic, and financial components, with 27 of 45 (60%) of the FP2020 country AY commitments receiving a score greater than 50% for completeness (Figure 1); this increased to 29 of 33 (88%) in the FP2030 country AY commitments. An examination of the policy scores shows that the number of country AY commitments with a policy component increased from 34 of 45 (76%) in FP2020 to 33 of 33 (100%) in FP2030 (Figure 2).

Two components of the programmatic score are service delivery and social behavior change. A comparison of scores for programmatic components shows that 40 of 45 (89%) of FP2020 AY country commitments included service delivery compared to 32 of 33 (97%) of FP2030 AY country commitments. For social and behavior change, the number of country commitments increased from 35 of 45 (78%) in FP2020 to 29 of 33 (88%) in FP2030.

We analyzed whether the country commitment specified that part of its financial commitment was dedicated to achieving AY commitments and if the amount of the budget or increase in budget is specified in the AY commitment. For example, Uganda’s FP2020 commitment included both increased financing for AYSRH (score 0.5) and specified 10% of the annual health sector budget (score 0.5). Malawi’s FP2030 commitment included increased financing for AY health services (score 0.5) but no specific amount (score 0). Of FP2020 countries, 5 of 45 (11%) included a budget for AY objectives compared to 5 of 33 (15%) of FP2030 countries (Figure 2). In terms of the specification of the amount of budget or increase in budget, 3 of 45 (7%) of FP2020 countries and 2 of 33 (6%) for FP2030 had scores of greater than 50%.

### Clarity

**FIGURE 3:**
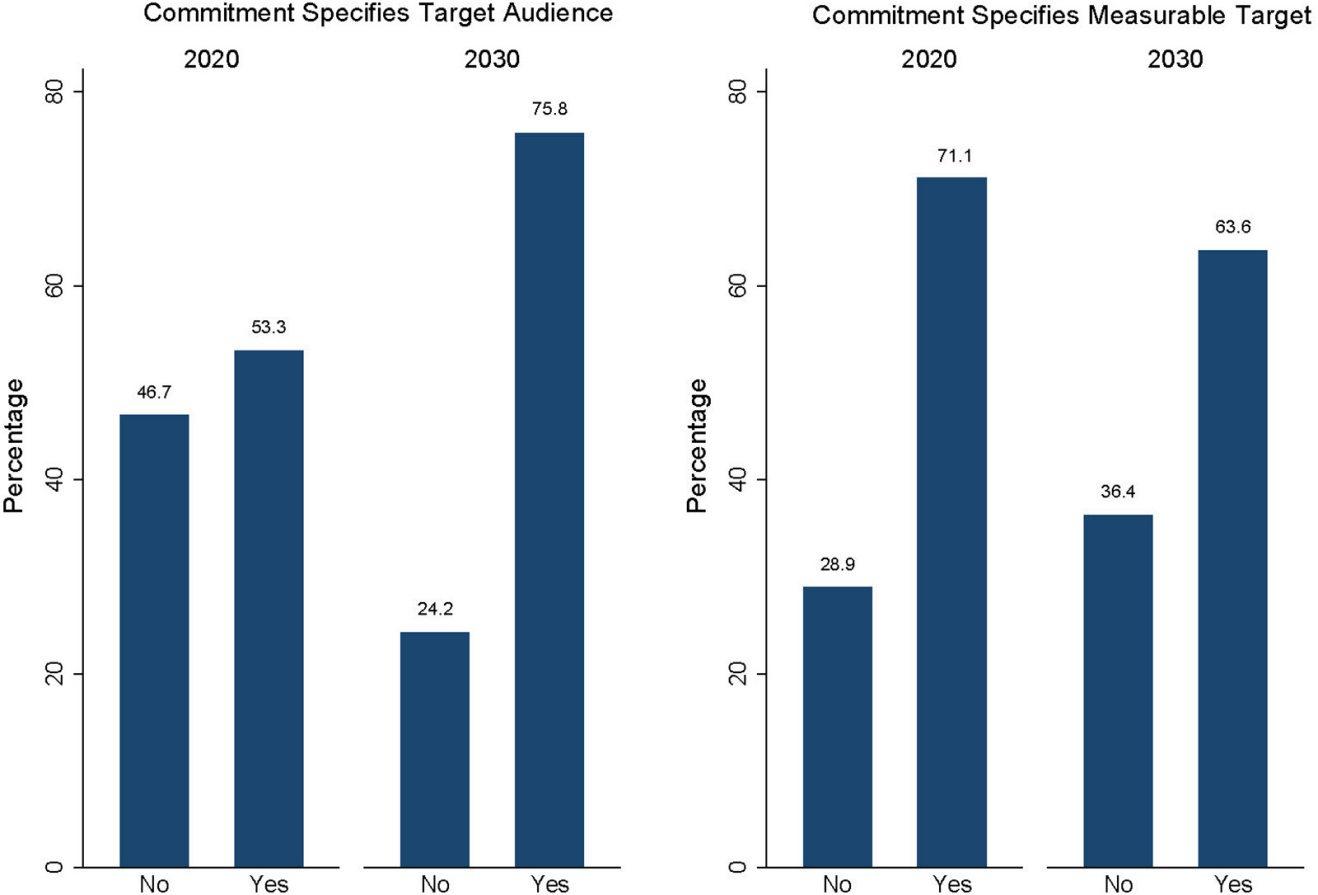
Clarity Domain Component Scores for Country Adolescent and Youth Commitments, FP2020 and FP2030

The clarity domain scores show whether AY country commitments were specific and clear, including information on the target audiences and whether they have measurable targets for monitoring the progress of their objectives. The AY country commitments with a clarity score over 50% of this domain score increased from 22 of 45 (49%) for FP2020 to 18 of 33 (55%) for FP2030 commitments (Figure 1). The country AY commitments with a specified target audience increased from 24 of 45 (53%) in FP2020 to 25 of 33 (76%) in FP2030 (Figure 3). For example, in FP2030, Bangladesh and Ghana specified married AY as their target audiences, and India specified boys and men. Chad, the Democratic Republic of the Congo, and Indonesia targeted parents, teachers, or health care providers. The number of country AY commitments with a measurable target for monitoring the progress of their objectives slightly decreased from 32 of 45 (71%) in FP2020 to 21 of 33 (64%) in FP2030 (Figure 3).

### Quality

**FIGURE 4:**
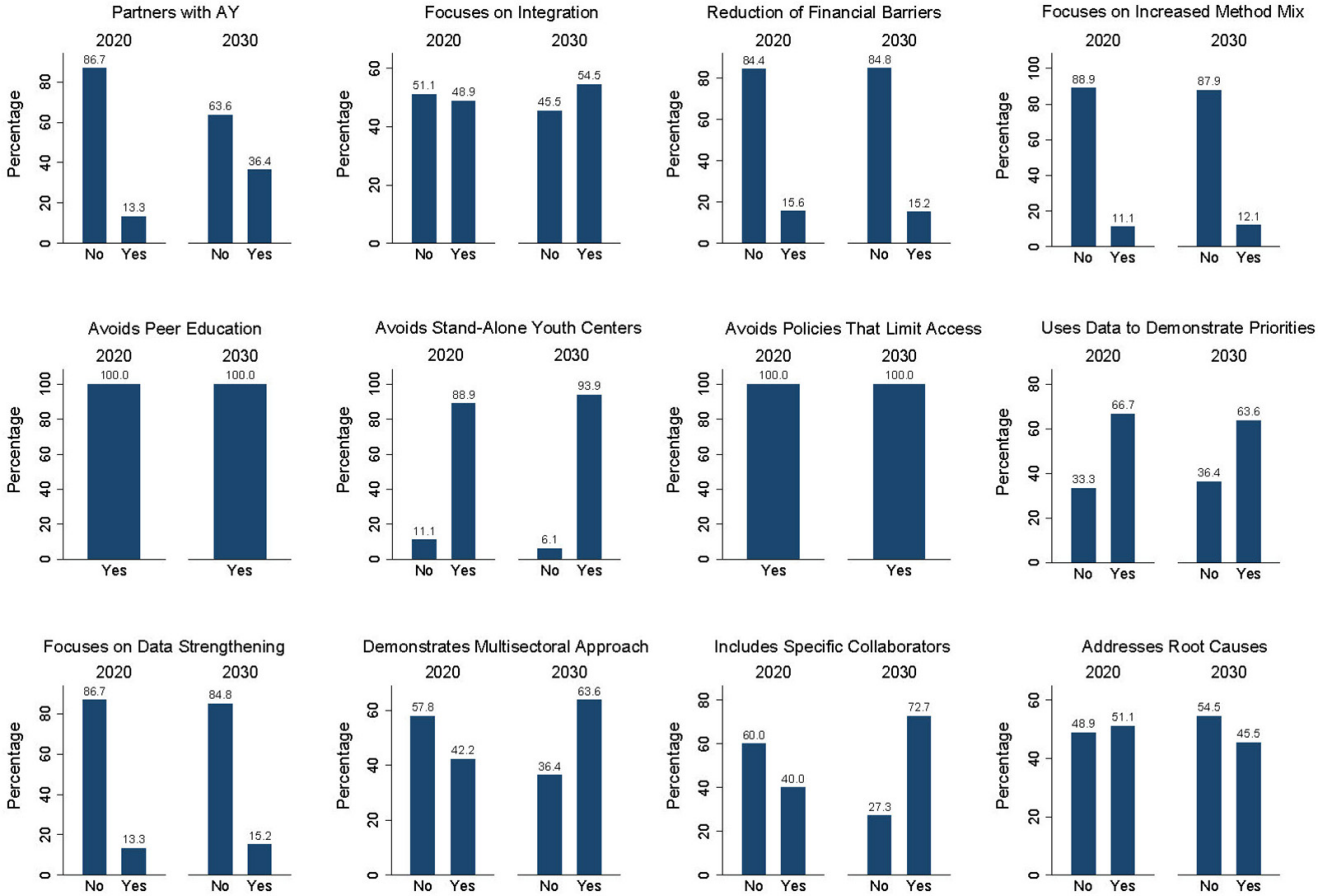
Quality Domain Component Scores for Country Adolescent and Youth Commitments, FP2020 and FP2030 Abbreviation: AY, adolescent and youth.

The quality domain scores measure whether AY country commitments included evidence-based and HIPs. These practices (e.g., partnership with AY or youth-led organizations, integration with other SRH interventions, and multisectoral approaches) are more likely to ensure positive outcomes for AYSRH. With respect to the quality of commitments, the countries scoring over 50% increased from 15 of 45 (33%) in FP2020 to 20 of 33 (60%) in FP2030 commitments (Figure 4).

We examined 6 components of quality. For the first component, which specified collaboration with AY, country commitments increased from 6 of 45 (13%) for FP2020 to 12 of 33 (36%) for FP2030. The second component referred to the use of evidence-based practices and HIPs that we measured in 7 subitems, including integrating FP within other SRH programs, reducing financial barriers, increasing the method mix, and using data. The other 3 subcomponents in this item consisted of non-implementation of practices that were not evidence based. The country AY commitments that included a focus on integrating FP within other SRH programs increased from 22 of 45 (49%) in FP2020 to 18 of 33 (55%) in FP2030. There was no substantial change between FP2020 country AY commitments that included a focus on reducing financial barriers to access at 7 of 45 (16%) compared to 5 of 33 (15%) of FP2030 country commitments. With respect to increasing the method mix for AY, there was no substantial change between FP2020 country AY commitments at 5 of 45 (11%) and FP2030 at 4 of 33 (12%). In terms of data use, the countries committing to use data to demonstrate AY FP priorities slightly decreased from 30 of 45 (67%) in 2020 to 21 of 33 (64%) in FP2030. The ineffective approach of stand-alone youth centers was excluded from 40 of 45 (89%) and 31 of 33 (94%) of FP2020 and FP2030 country AY commitments, respectively.

Our analysis showed that 6 of 45 (13%) of FP2020 country AY commitments included strengthening AY data compared to 5 of 33 (15%) in FP2030. The country AY commitments that included a multisectoral approach to AY increased from 19 of 45 (42%) in FP2020 to 21 of 33 (64%) in FP2030. In FP2020, 23 of 45 (51%) of country AY commitments identified root causes affecting adolescent and youth access to contraception and suggested interventions to tackle them. However, in FP2030, only 15 of 33 (46%) of country AY commitments did so, addressing issues like gender-based violence, violence against girls and women, child marriage, or girls’ education (Figure 4).

### Countries With Both FP2020 and FP2030 Commitments

We also analyzed and scored the 31 countries that have commitments in both FP2020 and FP2030 and include an AY component. While 16 of 31 (52%) of the FP2020 country AY commitments received a total score greater than 50% of the total possible domain score, for the FP2030 country AY commitments, this increased to 25 of 31 (81%) (Table 2). For the full sample, the total scores were 22 of 45 (49%) and 27 of 33 (82%) for FP2020 and FP2030 commitments, respectively (Figure 1). Completeness scores increased from 19 of 31 (61%) in FP2020 to 27 of 31 (87%) in FP2030. These findings are similar to the full sample that showed an increase from 27 of 45 (60%) to 29 of 33 (88%) over time (Figure 1). For the clarity domain, both in FP2020 and FP2030, this score remained at 17 of 31 (55%). However, in the full sample, the scores increased from 22 of 45 (49%) in FP2020 to 18 of 33 (55%) in FP2030 (Figure 1). In the quality domain, the 31 countries had an increase in this score from 11 (35%) in FP2020 to 19 (61%) in FP2030. These scores are remarkably similar to the scores for the full sample, which showed an increase from 15 of 45 (33%) to 20 of 33 (61%) from FP2020 to FP2030, respectively (Figure 1). The improvement over time in the country AY commitments was similar in trends to the full sample of 45 FP2020 commitments and 33 FP2030 commitments, except for clarity where there was no change.

**TABLE 2.** Countries With Both FP2020 and FP2030 Adolescent and Youth Commitments With a Score Greater Than 50 Percent of the Total Possible Domain Score

**(N=31)**	**Total, No. (%)**	**Completeness, No. (%)**	**Clarity, No. (%)**	**Quality, No. (%)**
**FP2020**	16 (52)	19 (61)	17 (55)	11 (35)
**FP2030**	25 (81)	27 (87)	17 (55)	19 (61)

## DISCUSSION

There has been substantial improvement in some aspects of completeness, clarity, and quality of country AY commitments from FP2020 to FP2030, but not in others. Over the period analyzed, there have been great strides in policies, partnering with AY or youth-led organizations, involving multiple sectors, and involving entities other than governmental sectors. We acknowledge that there is a slight increase in countries that included a budget for AY objectives in FP2030 commitments. However, even though most countries have made significant progress in AY commitments, they still lag in terms of having a specified budget for AY, measurable targets for monitoring the progress of their AY objectives, identifying and addressing root causes that impact AY, and clearly articulating their commitment to reducing financial barriers for AY.

There have been great strides in policies, partnering with AY or youth-led organizations, involving multiple sectors, and involving entities other than governmental sectors.

Over time, countries have developed a better understanding of effective strategies to improve access to, uptake, and sustained use of contraception for AY. Significant efforts have been made to stimulate and support countries to develop policies and strategies to improve access to and uptake of contraception by adolescents based on sound data, research evidence, and programmatic experience. Our analysis suggests that such efforts appear to have taken root in many countries, with many of them detailing tangible actions in their commitments that they will take to improve AYSRH outcomes. As mentioned in the introductory section, FP2020 and then FP2030 supported countries to develop commitments that were more inclusive of AY through a structured and ongoing process. It is likely that this assistance, in collaboration with its partners, has contributed to this progress. However, it is evident that this advancement cannot be solely attributed to FP2020 and FP2030.

Similar studies have been conducted by the Global Financing Facility, which was launched in 2015 by the World Bank to target the $US33 billion annual funding gap that hinders countries with serious reproductive, maternal, newborn, child, and adolescent health needs from meeting the 2030 Sustainable Development Goals.^26^ In a recent article analyzing 11 Global Financing Facility government planning documents and World Bank budgeted plans from 2018, researchers found that 5 countries invested positively in adolescent and youths’ sexual and reproductive health.^24^ However, similar to our results, there was less attention placed on investment compared to programming content, and very little attention was paid to addressing the social determinants that influence AY’s health.^26^ In our study, no FP2030 countries proposed ineffective approaches, such as stand-alone AY health services. Likewise, the Global Financing Facility study found that countries had progressed past ineffective solutions for SRH service delivery. In terms of child marriage, since the launch of the Global Programme on Child Marriage in 2016, the number of countries implementing a costed national strategy to end child marriage increased from 7 in 2018 to 33 in 2022.^27^ In our study, unfortunately, a lower number of FP2030 countries committed to addressing root causes including child marriage (15 of 33 countries, 46%), compared to FP2020 countries (23 of 45 countries, 51%). However, the Global Programme is seeing a consistent increase in both investment and programmatic focus on child marriage from their target countries.^27^ This calls for efforts to strengthen linkages between national efforts to end child marriage and respond to married girls and to improve access to, uptake, and continued use of contraception.^27^

Our findings have demonstrated both progress in including AY in country FP commitments as well as existing gaps in country plans. In terms of future action, an important follow-up action will be to support countries to examine and address areas of weakness in their plans. This study pointed to certain gaps such as the explicit statement of measurable targets to monitor and assess progress, increasing financing for AY programs, reducing financial barriers to contraception access for AY, and increasing the method mix.

This study illustrated certain gaps including having a measurable target to monitor and assess progress, increasing financing for AY programs, reducing financial barriers to contraception access for AY, and increasing the method mix.

Regarding research, an important follow-up study is an analysis of whether the commitments were, in fact, translated into action. This is eminently feasible given FP2030’s work with civil society organizations in countries and gathering self-reports from countries on the actions taken.

### Strengths and Limitations

One key strength of this study is that it compares 2 sets of commitments over a specific period of 10–12 years. Though the commitments are publicly available on the FP2030 website, our analysis of the commitments is based on a dataset that FP2020 began collecting in 2017 and completed in 2024.

Our study also uses an analytic framework that is in line with the guidance that countries were asked to use in developing their FP2030 commitments in 2020 and 2021. Finally, because of background documentation held by FP2030, information about the context in each country was available.

One limitation is that for some indicators, one needed to make educated guesses on a country’s intent in its commitment, as the language was not always precise. Our study looked at the content of the country commitments. The challenge now is to ensure countries have the resources and skills to implement their commitments and activities to support AY SRH.

## CONCLUSION

FP2020 signaled a stronger commitment to AY inclusion in national commitments on FP in 2016. Since then, there has been increased support to countries from multiple stakeholders to champion the importance of AYSRH, as well as increased assistance with the development of country commitments for AYSRH based on data, evidence from research, and continuing capacity support. This support to countries was provided through regional workshops, the participation of youth focal points beginning in 2019, and commitment guidance provided by FP2030’s AY commitment toolkit in 2020. Working as a broad alliance, staff in FP2020/FP2030, WHO, nongovernmental organizations working with and led by young people, and young people themselves have supported countries to develop complete, clearly articulated, and high-quality commitments to AYSRH as part of each country’s overall commitment, institutionalizing AYSRH as a primary strategy for policymakers and program managers to reduce unmet need and increase contraceptive use among young women and girls who would like to delay, space, or prevent pregnancy. While there have been improvements in AYSRH programming, our analysis shows that there is still room for improvement, particularly in areas of the root causes, including child marriage, and increased financial resources specifically for AYSRH programming.

## Supplementary Material

24-00223-Raney-Supplement.pdf

## Notes

**Peer Reviewed**

**First Published Online:** October 21, 2024.
