# Supplementary material for: FP2020 and FP2030 Country Commitments: A Mixed Method Study of Adolescent and Youth Sexual and Reproductive Health Components
Source: Glob Health Sci Pract. 2024 Oct 29;12(5):e2400223. doi: 10.9745/GHSP-D-24-00223 (PMC11521563; doi:10.9745/GHSP-D-24-00223)

Supplement to: Kamuyango A, Arora S, Raney L, Ali AK, Chandra-Mouli V. FP2020 and FP2030 country commitments: a mixed method study of adolescent and youth sexual and reproductive health components. *Glob Health Sci Pract*. 2024;12(5):2400223. https://doi.org/10.9745/GHSP-D-24-00223

Supplement Table S1. AY Country Commitment Scoring Guideline

**AY commitments scoring guideline**						
**Domain**	**Item**	**Definition**	**Scoring Indicator**		**Score**	**Domain score**
A) Completeness: AY commitments include policy, programmatic, and financial components	A.1) Policy commitments	*The country commits to establishing national or local policies, strategies, and guidance to achieve their objectives and to promote AY access to contraception, being more adolescent responsive, and achievement of better AY outcomes*	A.1.1) At least one AY commitment includes policy commitment(s)		1	3
	A.2.1) Programmatic commitments (Service delivery)	*The country commits to providing, improving, and upscaling AY services removing barriers to services to promote AY access to contraception and achieve their objectives*	A.2.1.1) At least one AY commitment includes service delivery component(s)		0.5	
	A.2.2) Programmatic commitments (Social and behavioral change)	*The country commits to implementing interventions to address individual, interpersonal, and community level factors and to achieve its objectives, and enable better AY access to and use of contraception*	A.1.2.2) At least one AY commitment includes SBC component(s)		0.5	
	A.3) Financial commitments	*The country commits to dedicating part of its budget to enable the success of their objectives and promote AY contraception, uptake of contraception, and access to services*	A.3.1) AY commitments dedicate a budget for achieving AY objectives		0.5	
			A.3.2) The amount of the budget for AY - or budget increase for AY - is specified in the AY commitment		0.5	
B) Clarity: AY commitments are specific and clear	B.1) Target audience of the commitments	*The AY commitments clearly specify the target audience of their objectives*	B.1.1) At least one AY commitment specifies the target audience of	B.1.1.1) Married	0.5	1
				B.1.1.2) Unmarried		
				B.1.1.3) Boys and/or Men		

Supplement to: Kamuyango A, Arora S, Raney L, Ali AK, Chandra-Mouli V. FP2020 and FP2030 country commitments: a mixed method study of adolescent and youth sexual and reproductive health components. *Glob Health Sci Pract*. 2024;12(5):2400223. https://doi.org/10.9745/GHSP-D-24-00223

AY commitments scoring guideline

Domain	Item	Definition	Scoring Indicator		Score	Domain score
			their objectives	B.1.1.4) Others: Parents, Partners, Teachers, Healthcare Providers… etc. (Just mark 'others'.		
	B.2) Measurement of the commitments	*The AY commitment includes a measurable target for monitoring progress of their objectives*	B.2.1) At least one AY commitment includes a measurable target		0.5	
C) Quality: AY commitments address considerations that could contribute to the achievement or success of the commitments	C.1) Involvement of AY in commitment objectives	*The AY commitment highlights partnership with AY or youth- led organizations, and considers jointly developing, launching, and/or implementing the commitment and achievement of the commitment objectives*	C.1.1) At least one AY commitment illustrates partnering with AY or youth-led organizations		1	9
	C.2) Use of evidence and data-based approaches	*The AY commitments use data-based approaches, including data disaggregation by age, gender and other factors- to determine their objectives, in addition to using evidence-based approaches such as high impact practices, and avoiding the use ineffective interventions or strategies in their objectives*	C.2.1.1) An AY commitment focuses on integration of FP and contraception for adolescents within SRH, HIV, immunization, postpartum family planning, or post-abortion family planning services		0.5	
			C.2.1.2) An AY commitment focuses on reducing financial barriers to access such as providing free access to contraception, establishing a subsidiary mechanism, applying for funding, etc.		0.5	
			C.2.1.3) An AY commitment focuses on increasing the method mix choice		0.5	

Supplement to: Kamuyango A, Arora S, Raney L, Ali AK, Chandra-Mouli V. FP2020 and FP2030 country commitments: a mixed method study of adolescent and youth sexual and reproductive health components. *Glob Health Sci Pract*. 2024;12(5):2400223. https://doi.org/10.9745/GHSP-D-24-00223

<table>
<tr><th colspan="7">AY commitments scoring guideline</th></tr>
<tr><th>Domain</th><th>Item</th><th>Definition</th><th colspan="2">Scoring Indicator</th><th>Score</th><th>Domain score</th></tr>
<tr><td rowspan="10"></td><td rowspan="4"></td><td rowspan="4"></td><td colspan="2">C.2.2.1) AY commitments avoid the use of standalone peer education and do not mention additional strategies or interventions</td><td>0.5</td><td rowspan="10"></td></tr>
<tr><td colspan="2">C.2.2.2) AY commitments avoid the use of standalone youth centers or clinics</td><td>0.5</td></tr>
<tr><td colspan="2">C.2.2.3) AY commitments avoid policies or programmes that limit access of specific groups of adolescents, based on their age or gender</td><td>0.5</td></tr>
<tr><td colspan="2">C.2.3) At least one AY commitment uses data to demonstrate priority issues, groups, or interventions</td><td>1</td></tr>
<tr><td>C.3) Strengthening data quality</td><td>The AY commitments focus on enhancing the country's capacity to collect and utilize disaggregated data by age, gender and other factors to prioritize AY issues or AY target audiences, measure the commitments' progress or evaluate their objectives</td><td colspan="2">C.3.1) At least one AY commitment includes an objective of strengthening data mechanisms to use disaggregated data in their operations such as integrating data disaggregation in HMIS, disaggregating indicators used in surveys, among others</td><td>1</td></tr>
<tr><td>C.4) Use of Multisectoral approaches</td><td>The AY commitments involve multiple sectors within the government that are concerned with achieving the commitments objectives</td><td colspan="2">C.4.1) At least one AY commitment includes an objective where it demonstrates a multisectoral approach</td><td>1</td></tr>
<tr><td rowspan="4">C.5) Commitments involve entities other the governmental sectors</td><td rowspan="4">The AY commitments involve entities other than governmental sectors that are concerned with achieving the commitments objectives</td><td rowspan="4">C.5.1) At least one AY commitment includes an objective where it one of the implementers or collaborators has one or</td><td>C.5.1.1) NGOs or CBOs</td><td rowspan="4">1</td></tr>
<tr><td>C.5.1.2) Private sector</td></tr>
<tr><td>C.5.1.3) Donors</td></tr>
<tr><td>C.5.1.4) Academic institutes</td></tr>
</table>

Supplement to: Kamuyango A, Arora S, Raney L, Ali AK, Chandra-Mouli V. FP2020 and FP2030 country commitments: a mixed method study of adolescent and youth sexual and reproductive health components. *Glob Health Sci Pract*. 2024;12(5):2400223. https://doi.org/10.9745/GHSP-D-24-00223

AY commitments scoring guideline						
**Domain**	**Item**	**Definition**	**Scoring Indicator**		**Score**	**Domain score**
			more of the following entities	C.5.1.5) Other non-governmental sector		
	C.6) Commitments address root causes	*The AY commitments highlight root causes that impacts AY access to and use of contraception, and propose measures to address these root causes*	C.6.1) At least one AY commitment addresses root causes such GVB, VAGW, Child Marriage, or Girls' Education		1	
Total score					13	

Supplement to: Kamuyango A, Arora S, Raney L, Ali AK, Chandra-Mouli V. FP2020 and FP2030 country commitments: a mixed method study of adolescent and youth sexual and reproductive health components. *Glob Health Sci Pract*. 2024;12(5):2400223. https://doi.org/10.9745/GHSP-D-24-00223

Supplement Table S2. Countries that made a FP2020 commitment, FP2030 commitment, or both. "X" signifies that the country made a commitment.

Country	FP2020	FP2030
Angola	x	
Afghanistan	x	
Bangladesh	x	x
Benin	x	x
Burkina Faso		x
Burundi	x	x
Cameroon	x	
Central African Rep	x	
Chad	x	x
Cote d'Ivoire	x	x
DRC	x	x
Ethiopia	x	x
Ghana	x	x
Guinea	x	x
Haiti	x	
India	x	x
Indonesia	x	x
Kenya	x	x
Kyrgyz Republic	x	x
Lao Republic	x	
Liberia	x	
Madagascar	x	x
Malawi	x	x
Mali	x	x
Mauritania	x	x
Mozambique	x	x
Myanmar	x	
Namibia		x
Nepal	x	x
Niger	x	x
Nigeria	x	x
Pakistan	x	x
Philippines	x	x
Rwanda	x	x

Supplement to: Kamuyango A, Arora S, Raney L, Ali AK, Chandra-Mouli V. FP2020 and FP2030 country commitments: a mixed method study of adolescent and youth sexual and reproductive health components. *Glob Health Sci Pract*. 2024;12(5):2400223. https://doi.org/10.9745/GHSP-D-24-00223

**Country**	**FP2020**	**FP2030**
Senegal	x	x
Sierra Leone	x	
Solomon Islands	x	
Somalia	x	
South Sudan	x	x
Sri Lanka	x	
Tanzania	x	x
The Gambia	x	
Togo	x	x
Uganda	x	x
Vietnam	x	
Zambia	x	x
Zimbabwe	x	x
**Total**	**45**	**33**

Burkina Faso was not added for FP2020 analysis because despite having a commitment, there was no adolescent youth content to be analyzed.

Supplement to: Kamuyango A, Arora S, Raney L, Ali AK, Chandra-Mouli V. FP2020 and FP2030 country commitments: a mixed method study of adolescent and youth sexual and reproductive health components. *Glob Health Sci Pract*. 2024;12(5):2400223. https://doi.org/10.9745/GHSP-D-24-00223

Supplement Figure S1. Completeness of Country AY Commitments, FP2020

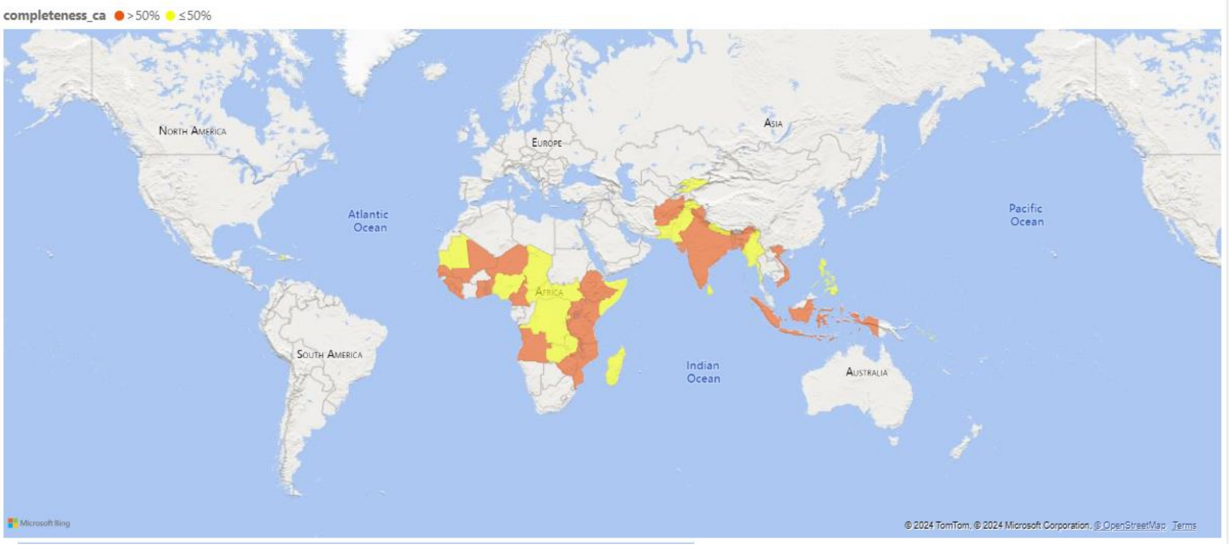

Supplement Figure S2. Completeness of Country AY Commitments, FP2030

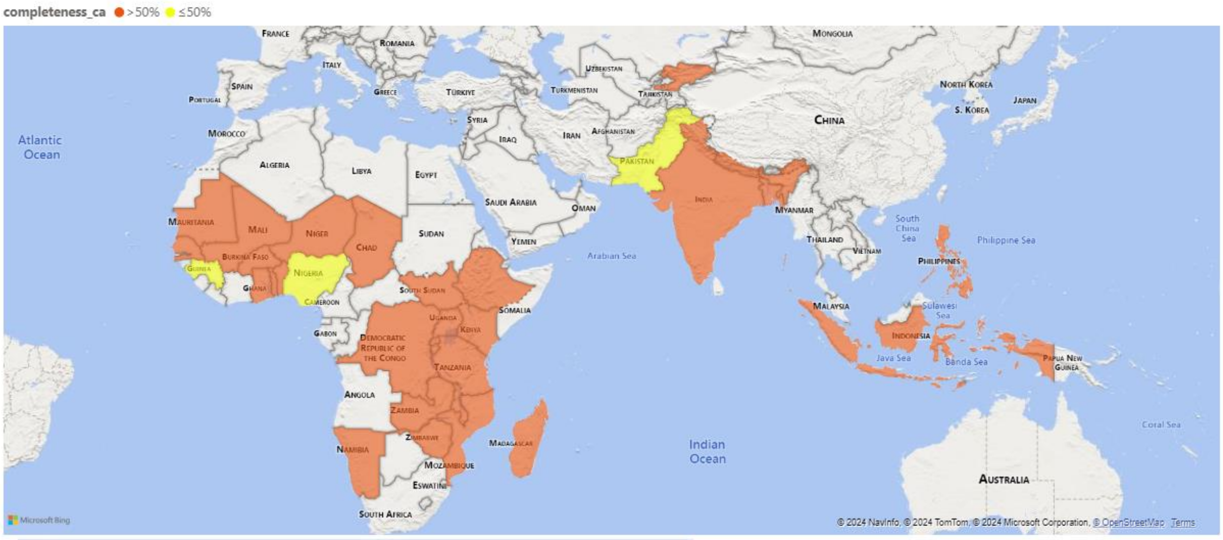

Supplement to: Kamuyango A, Arora S, Raney L, Ali AK, Chandra-Mouli V. FP2020 and FP2030 country commitments: a mixed method study of adolescent and youth sexual and reproductive health components. *Glob Health Sci Pract*. 2024;12(5):2400223. https://doi.org/10.9745/GHSP-D-24-00223

Supplement Figure S3. Quality of Country AY Commitments, FP2020

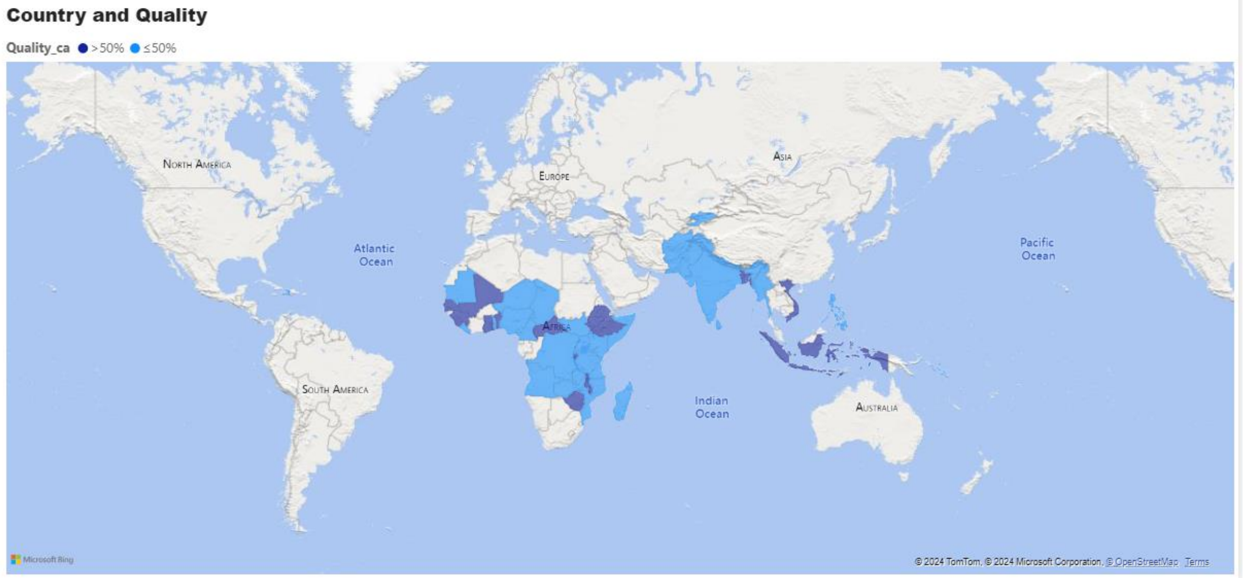

Supplement Figure S4. Quality of Country AY Commitments, FP2030

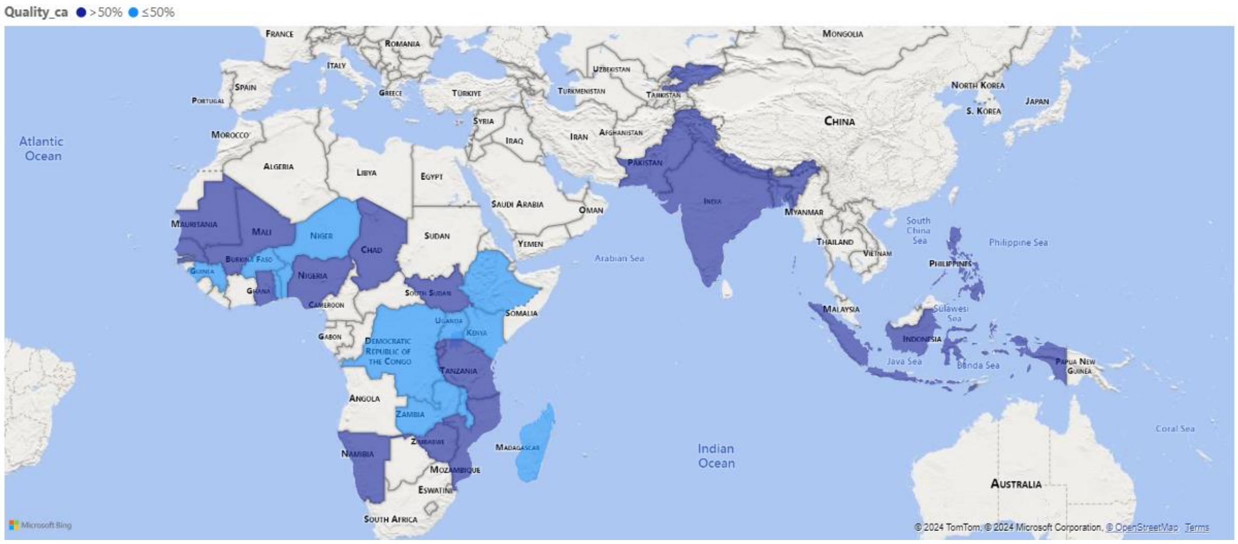

Supplement to: Kamuyango A, Arora S, Raney L, Ali AK, Chandra-Mouli V. FP2020 and FP2030 country commitments: a mixed method study of adolescent and youth sexual and reproductive health components. *Glob Health Sci Pract*. 2024;12(5):2400223. https://doi.org/10.9745/GHSP-D-24-00223

Supplement Figure S5. Clarity of Country AY Commitments, FP2020

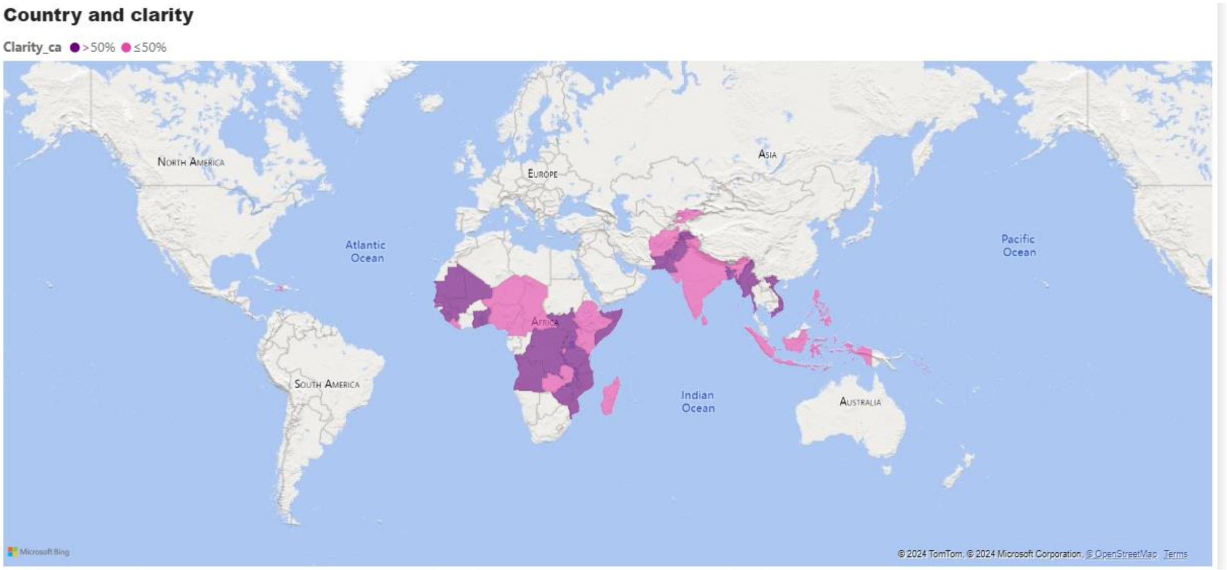

Supplement Figure S6. Clarity of Country AY Commitments, FP2030

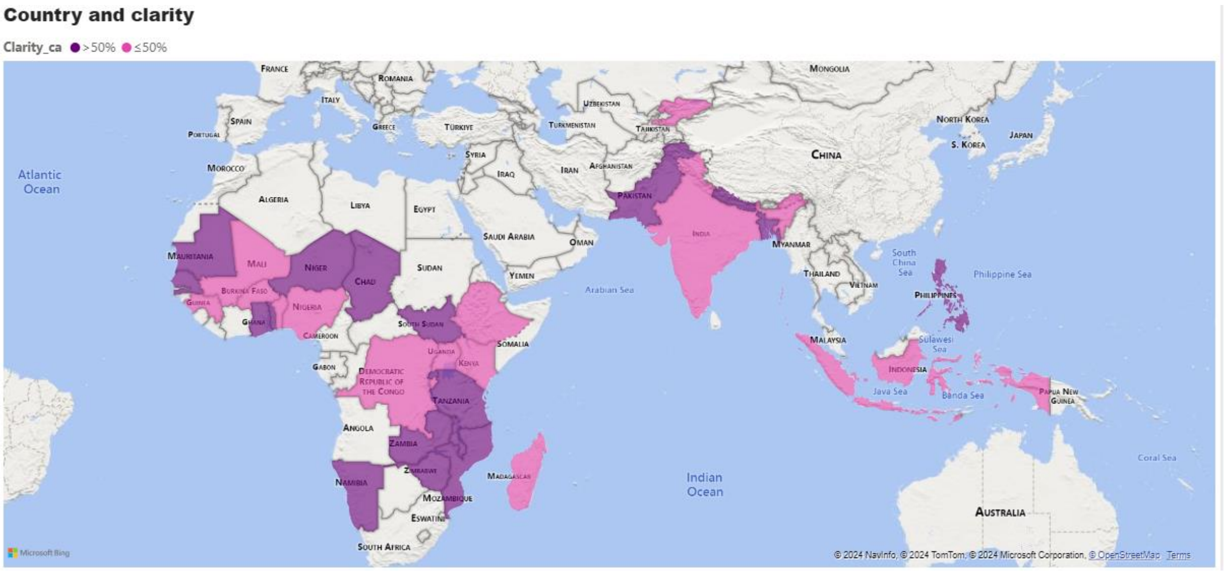

Supplement: 24-00223-Raney-Supplement.pdf [file 24-00223-Raney-Supplement.pdf]
